# Pentoxifylline Effects on Nerve Conduction Velocity and Blood Flow in Diabetic Rats

**DOI:** 10.1155/EDR.2000.49

**Published:** 2000

**Authors:** Heather Flint, Mary A. Cotter, Norman E. Cameron

**Affiliations:** 1 Department of Biomedical Sciences Institute of Medical Sciences University of Aberdeen Foresterhill, Aberdeen Scotland AB25 2ZD United Kingdom

## Abstract

Pentoxifylline has several actions that improve blood rheology and tissue perfusion and may therefore potentially be applicable to diabetic neuropathy. The aims of this study were to ascertain whether 2 weeks of treatment with pentoxifylline could correct nerve conduction velocity and blood flow deficits in 6-week streptozotocin-diabetic rats and to examine whether the effects were blocked by co-treatment with the cyclooxygenase inhibitor, flurbiprofen, or the nitric oxide synthase inhibitor, N^G^-nitro-ʟ-arginine. Diabetic deficits in sciatic motor and saphenous sensory nerve conduction velocity were 56.5% and 69.8% corrected, respectively, with pentoxifylline treatment. Sciatic endoneurial blood flow was approximately halved by diabetes and this deficit was 50.4% corrected by pentoxifylline. Flurbiprofen co-treatment markedly attenuated these actions of pentoxifylline on nerve conduction and blood flow whereas N^G^-nitro-ʟ-arginine was without effect. Thus, pentoxifylline treatment confers neurovascular benefits in experimental diabetic neuropathy, which are linked at least in part to cyclooxygenasemediated metabolism.


*Int. Jnl. Experimental Diab. Res.*, Vol. 1, pp. 49–58
Reprints available directly from the publisher
Photocopying permitted by license only

© 2000 OPA (Overseas Publishers Association) N.V.
Published by license under
the Harwood Academic Publishers imprint,
part of The Gordon and Breach Publishing Group.
Printed in Malaysia.

### Pentoxifylline Effects on Nerve Conduction Velocity and Blood Flow in Diabetic Rats

HEATHER FLINT, MARY A. COTTER and NORMAN E. CAMERON*

*Department of Biomedical Sciences, Institute of Medical Sciences, University of Aberdeen, Foresterhill, Aberdeen AB25 2ZD, Scotland UK*

*(Received 16 September 1999; Revised 27 October 1999; In final form 29 October 1999)*

**Pentoxifylline has several actions that improve blood rheology and tissue perfusion and may therefore potentially be applicable to diabetic neuropathy. The aims of this study were to ascertain whether 2 weeks of treatment with pentoxifylline could correct nerve conduction velocity and blood flow deficits in 6-week streptozotocin-diabetic rats and to examine whether the effects were blocked by co-treatment with the cyclooxygenase inhibitor, flurbiprofen, or the nitric oxide synthase inhibitor, $N^G$-nitro-L-arginine. Diabetic deficits in sciatic motor and saphenous sensory nerve conduction velocity were 56.5% and 69.8% corrected, respectively, with pentoxifylline treatment. Sciatic endoneurial blood flow was approximately halved by diabetes and this deficit was 50.4% corrected by pentoxifylline. Flurbiprofen co-treatment markedly attenuated these actions of pentoxifylline on nerve conduction and blood flow whereas $N^G$-nitro-L-arginine was without effect. Thus, pentoxifylline treatment confers neurovascular benefits in experimental diabetic neuropathy, which are linked at least in part to cyclooxygenase-mediated metabolism.**

*Keywords:* Diabetes, neuropathy, rat, blood flow, ischemia, nerve conduction, phosphodiesterase inhibitor, vasodilation

### INTRODUCTION

The early neuropathic changes in experimental diabetes, such as diminished conduction velocity (NCV), are believed to be largely attributable to a reduction in nerve perfusion (Tuck *et al.*, 1984; Cameron *et al.*, 1991). Parallels may be drawn with reduced sural nerve blood flow and the presence of endoneurial hypoxia in neuropathic patients (Tesfaye *et al.*, 1994). Several vasodilators have been identified that partially prevent or correct nerve dysfunction in diabetic rats (Cameron *et al.*, 1994a), and patients (Tesfaye *et al.*, 1994), presumably by improving perfusion and oxygenation. Major contributions to impaired nerve blood flow are made by decreased vasa nervorum prostacyclin and nitric oxide (NO) production or action, and elevated vasoconstrictor activity (Ward *et al.*, 1989; Kihara and Low, 1995; Maxfield *et al.*,

*Corresponding author. Tel.: +44 1224 273013, Fax: +44 1224 273019, e-mail: n.e.cameron@abdn.ac.uk

1995, 1997; Cameron and Cotter, 1996a). In turn, these changes result from metabolic consequences of hyperglycemia, including increased polyol pathway, elevated production of reactive oxygen species, advanced glycation and protein kinase C activation (Cameron *et al.*, 1994a, 1994b, 1996; Cameron and Cotter, 1996b; Cameron *et al.*, 1999; Sima and Sugimoto, 1999). Diabetes also causes elevated blood viscosity, decreased red cell deformability, increased platelet activation, a prothrombotic state, and phagocyte activation that may contribute to reduced nerve perfusion (Simpson, 1988; Ceriello, 1993).

Pentoxifylline is a methylxanthine derivative possessing hemorheological actions that improve the microcirculation (Ward and Clissold, 1987). It is a phosphodiesterase inhibitor that can cause vasodilation in some vessels by endothelium-dependent and independent mechanisms (Hansen, 1994; Kaputlu and Sadan, 1994). Pentoxifylline also inhibits production of platelet activating factor, reduces platelet aggregation, increases red cell membrane fluidity, and reduces the production of inflammatory cytokines, particularly tumor necrosis factor, by phagocytes and vascular endothelium (Adams *et al.*, 1995; Ambrus *et al.*, 1995; Bernard *et al.*, 1995; Mandell, 1995). Thus, pentoxifylline has vascular and rheological actions that may counteract some of the changes in diabetes that contribute to nerve dysfunction. The aim of this study was to determine whether pentoxifylline treatment corrects NCV and perfusion deficits in diabetic rats. As some putative actions of pentoxifylline involve correction or potentiation of the effects of prostacyclin and NO systems, the outcomes of co-treatment with the cyclooxygenase inhibitor, flurbiprofen, or the NO synthase inhibitor, $N^G$-nitro-L-arginine, were examined to further elucidate underlying mechanisms. Preliminary data were presented at a meeting of the International Diabetes Federation (Cotter *et al.*, 1997).

### MATERIALS AND METHODS

Male Sprague-Dawley rats, 19 weeks of age at the start of the experiment, were used. Diabetes was induced by intraperitoneal injection (40–45 mg $kg^{-1}$) of streptozotocin (Zeneca, Macclesfield, Cheshire, UK) freshly made up in sterile saline. Diabetes was verified 24 h later by estimating hyperglycemia and glycosuria (Visidex II and Diastix; Ames, Slough, UK). Rats were tested weekly, and weighed daily; they were rejected if blood glucose was $<20$ mM or if they showed a consistent increase in body weight over 3 days. Samples for plasma glucose measurement (GOD-Perid method; Boehringer, Mannheim, Germany) were taken from the tail vein before or from a carotid cannula after final experiments.

Experimental groups comprised non-diabetic onset control rats ($n=20$), 8-week untreated diabetic rats ($n=20$), and 6-week untreated diabetic groups treated for a further 2 weeks with pentoxifylline (Sigma, Poole, Dorset, UK) at a dose of 40 mg $kg^{-1}$ $day^{-1}$ p.o. ($n=26$) or pentoxifylline + $N^G$-nitro-L-arginine (Sigma; 10 mg $kg^{-1}$ $day^{-1}$, added to the drinking water) ($n=22$) or pentoxifylline + flurbiprofen (Sigma; 5 mg $kg^{-1}$ $day^{-1}$, added to the drinking water) ($n=20$). Doses of $N^G$-nitro-L-arginine and flurbiprofen were chosen to have modest effects on neurovascular function in non-diabetic rats, but to be effective in blocking effects of certain interventions in diabetic rats, including aldose reductase inhibition and $\omega$-6 essential fatty acid treatment (Cameron *et al.*, 1996, 1993). Separate groups of rats were used to assess motor and sensory NCV ($n=10-16$), and sciatic nerve perfusion ($n=9-10$).

At the end of the treatment period, rats were anesthetized with urethane (Sigma; 1–1.5 mg $kg^{-1}$), or for the nerve perfusion experiments with thiobutabarbital (Zeneca; 50–100 mg $kg^{-1}$), by intraperitoneal injection. The trachea was cannulated for artificial ventilation. A carotid cannula was used to monitor mean systemic blood pressure in rats undergoing blood flow measurement. Motor NCV was assessed between the sciatic notch and the knee in the nerve branch innervating the tibialis anterior muscle as previously described (Cameron *et al.*, 1989). This is representative of the whole sciatic nerve in terms of susceptibility to diabetes and treatment effects. Sensory NCV was measured in saphenous nerve between the groin and the ankle as previously described (Cameron *et al.*, 1989). Rectal and nerve temperatures were monitored, and kept between 36.5 and 37.5°C.

Sciatic endoneurial blood flow was measured by microelectrode polarography and hydrogen clearance as previously described (Cameron *et al.*, 1991). Briefly, the core temperature of the rat was monitored and kept between 37 and 38°C, using a rectal probe and radiant heat. The skin around the sciatic nerve incision was sutured to a metal ring and used to form a pool, which was filled with mineral oil. Pool temperature was maintained between 35 and 37°C by radiant heat during blood flow measurements. Rats were given neuromuscular blockade using d-tubocurarine (Sigma, 2 mg $kg^{-1}$ *via* the carotid cannula) and were artificially ventilated. The level of anesthesia was monitored by observing blood pressure reactions to manipulations and supplementary anaesthetic was given as necessary. A glass-insulated platinum microelectrode was inserted into the middle portion of the sciatic nerve, above its trifurcation, and polarized at 0.25 V with respect to a subcutaneous reference electrode. 10% $H_2$ was added to the inspired gas, the proportions of $O_2$ and $N_2$ being adjusted to 20% and 70% respectively. When the $H_2$ current recorded by the electrode had stabilized, indicating equilibrium with arterial blood, the $H_2$ supply was shut off and $N_2$ delivery was increased appropriately. The $H_2$ clearance curve was recorded until baseline values were reached, the latter being defined as no systematic decline in electrode current over 5 min. This procedure was then repeated at another site of the sciatic nerve. After the experiment, clearance curves were digitized and mono-exponential or bi-exponential curves were fitted to the data by computer using non-linear regression software that employs the Marquardt algorithm and the least squares method for optimizing goodness-of-fit (Prism, Graphpad, San Diego, CA, USA). The slow exponent was taken to reflect nutritive (capillary) flow (Day *et al.*, 1989). Vascular conductance was calculated by dividing flow by the mean arterial blood pressure during the recording period. The average of the two determinations was taken as representative for all measures of sciatic endoneurial blood flow.

### Statistical Analysis

Data are presented as group means ± SEM. They were given Bartlett's test for homogeneity of variances, followed by log transformation where appropriate (nutritive vascular conductance and composite blood flow) before being subjected to one-way analysis of variance. When overall significance ($P < 0.05$) was attained, between-group differences were established by post hoc analysis using the Student-Newman-Keuls test which is corrected for multiple comparisons.

### RESULTS

Diabetic rats showed an approximately 5-fold increase in plasma glucose concentration (Tab. I) compared to controls and had an approximately 27% body weight loss over the 8-week experimental period. These parameters were not significantly altered by pentoxifylline, $N^G$-nitro-L-arginine or flurbiprofen treatment.

Sciatic motor NCV (Fig. 1A) was 20.6 ± 0.9% reduced by 8 weeks of diabetes. Pentoxifylline treatment for the last 2 weeks corrected this NCV deficit by 56.5 ± 7.7% ($P < 0.001$), although conduction remained reduced compared to the

TABLE I Body weights and plasma glucose concentrations

| Group | *n* | Body weight(g) | Glucose (mM) |
|---|---|---|---|
| Non-diabetic | 20 | 435 ± 5 | 8.3 ± 0.5 |
| Diabetic | 20 | 313 ± 9 | 45.0 ± 1.8 |
| + pentoxifylline | 26 | 334 ± 7 | 39.8 ± 1.4 |
| + pentoxifylline + $N^G$-nitro-L-arginine | 22 | 310 ± 7 | 40.0 ± 1.4 |
| + pentoxifylline + flurbiprofen | 20 | 314 ± 8 | 41.4 ± 1.4 |

Data are mean ± SEM.

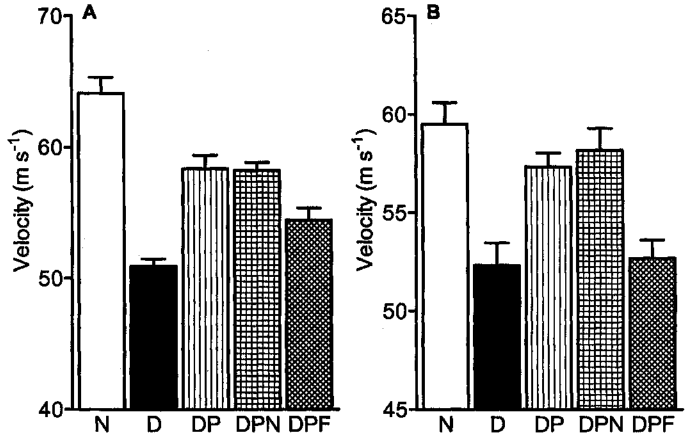

FIGURE 1 Effects of diabetes and treatment with pentoxifylline, alone and in combination with nitric oxide synthase or cyclooxygenase inhibition, on (A) sciatic nerve motor and (B) saphenous nerve sensory conduction velocity. N, non-diabetic controls ($n = 10$); D, 8-week diabetic controls ($n = 10$); DP, 8-week diabetic rats treated for the last 2 weeks with 40 mg $kg^{-1}$ $day^{-1}$ pentoxifylline ($n = 16$); DPN, 8-week diabetic rats treated for 2 weeks with pentoxifylline and 10 mg $kg^{-1}$ $day^{-1}$ $N^G$-nitro-L-arginine ($n = 13$); DPF, 8-week diabetic rats treated for 2 weeks with pentoxifylline and 5 mg $kg^{-1}$ $day^{-1}$ flurbiprofen ($n = 11$). Data are mean ± SEM.

non-diabetic group ($P < 0.001$). Co-treatment with $N^G$-nitro-L-arginine did not significantly alter the effect of pentoxifylline (55.4 ± 4.6% correction; $P < 0.001$ *versus* diabetic and non-diabetic groups). In contrast, flurbiprofen attenuated pentoxifylline's action by 52.9 ± 12.6% ($P < 0.01$), although the resultant motor NCV value remained significantly ($P < 0.05$) greater than that of the diabetic control group.

Sensory saphenous NCV (Fig. 1B) was 12.1 ±1.9% ($P < 0.001$) reduced by untreated diabetes. This was completely corrected by pentoxifylline treatment ($P < 0.01$). $N^G$-nitro-L-arginine co-treatment was without effect on pentoxifylline's action; NCV was in the non-diabetic range. However, with flurbiprofen co-treatment, NCV remained at the untreated diabetic level ($P < 0.01$ *versus* pentoxifylline treatment alone).

A 48.4 ± 2.2% diabetic deficit ($P < 0.001$) in sciatic nutritive endoneurial blood flow (Fig. 2A) was partially (44.7 ± 8.4%; $P < 0.001$) corrected by pentoxifylline treatment, although a significant deficit remained compared to the non-diabetic control group ($P < 0.001$). The effect of pentoxifylline was not significantly attenuated by $N^G$-nitro-L-arginine co-treatment, but it was 84.0 ± 4.0% diminished ($P < 0.05$) by flurbiprofen such that the resultant value was in the upper diabetic range.

Mean systemic blood pressure (Fig. 2B) tended to be reduced in the diabetic groups, although this was only statistically significant ($P < 0.05$) for untreated diabetes. Within the diabetic groups, the highest pressures were recorded with $N^G$-nitro-L-arginine co-treatment. As sciatic vasa nervorum does not show

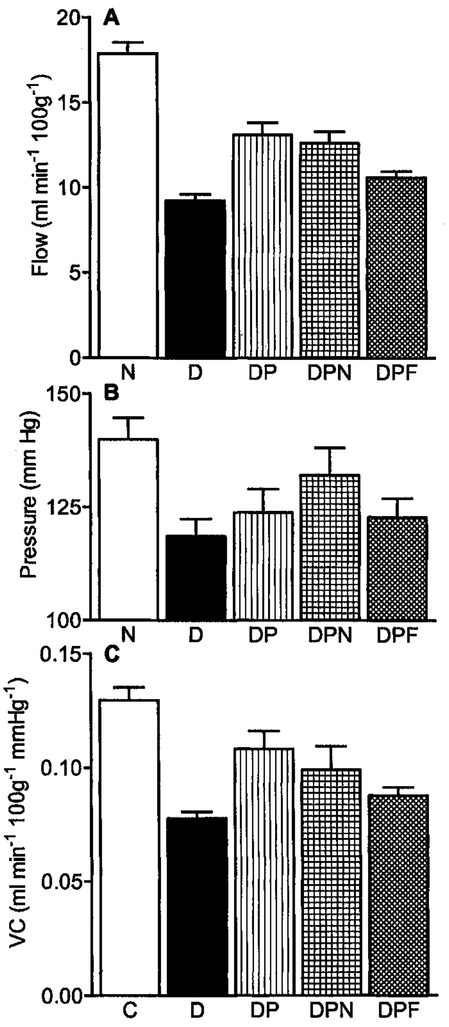

FIGURE 2 Effects of diabetes and treatment with pentoxifylline, alone and in combination with nitric oxide synthase or cyclooxygenase inhibition, on (A) sciatic nutritive endoneurial blood flow, (B) mean systemic blood pressure and (C) nutritive endoneurial vascular conductance (VC). N, non-diabetic controls ($n = 10$); D, 8-week diabetic controls ($n = 10$); DP, 8-week diabetic rats treated for the last 2 weeks with $40\,mg\,kg^{-1}\,day^{-1}$ pentoxifylline ($n = 10$); DPN, 8-week diabetic rats treated for 2 weeks with pentoxifylline and $10\,mg\,kg^{-1}\,day^{-1}$ $N^G$-nitro-L-arginine ($n = 9$); DPF, 8-week diabetic rats treated for 2 weeks with pentoxifylline and $5\,mg\,kg^{-1}\,day^{-1}$ flurbiprofen ($n = 9$). Data are mean ± SEM.

appreciable pressure autoregulation (Day *et al.*, 1989) flow results are expressed as vascular conductance (Fig. 2C) to take account of these pressure differences. A 40.0 ± 2.3% diabetic conductance deficit ($P < 0.001$) was 58.9 ± 15.1% ($P < 0.01$) corrected by pentoxifylline treatment. This was 29.8% and 66.9% attenuated by $N^G$-nitro-L-arginine and flurbiprofen co-treatments respectively, although in neither case did the effect reach statistical significance. While conductances for all treated diabetic rats remained below ($P < 0.01$) that of the non-diabetic group, the value for the pentoxifylline+$N^G$-nitro-L-arginine group exceeded that of untreated diabetes ($P < 0.05$) whereas this was not the case for pentoxifylline + flurbiprofen treated rats.

The hydrogen clearance microelectrode polarography method records both nutritive (capillary) endoneurial and non-nutritive (large vessel and arterio-venous anastomotic) flow (Cameron *et al.*, 1996; Day *et al.*, 1989). The weighted sum of these components describes total or composite endoneurial flow (Fig. 3A). This was 58.3 ± 3.3% ($P < 0.001$) reduced by diabetes and 55.6 ± 14.0% ($P < 0.01$) corrected by pentoxifylline treatment, the resultant value being not significantly different from that of the non-diabetic control group. Both $N^G$-nitro-L-arginine ($P < 0.01$) and flurbiprofen ($P < 0.001$) co-treatments completely attenuated the effects of pentoxifylline on composite endoneurial blood flow. Similar effects were seen for composite vascular conductance (Fig. 3B), the value for pentoxifylline treatment alone being in the non-diabetic range whereas those for $N^G$-nitro-L-arginine and flurbiprofen co-treatment were in the diabetic range. The percentage of hydrogen clearance carried by nutritive flow (Fig. 3C) was not significantly affected by diabetes. However, with pentoxifylline treatment alone, this was approximately halved compared to all the other groups ($P < 0.05$).

### DISCUSSION

The data show that pentoxifylline treatment partially corrected sciatic motor and completely corrected saphenous sensory NCV. Greater responsiveness of sensory conduction deficits

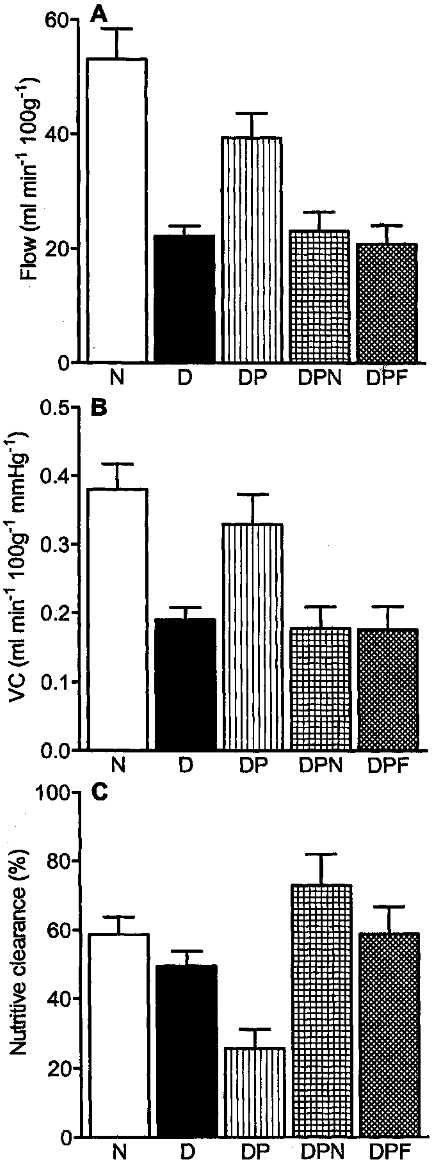

FIGURE 3 Effects of diabetes and treatment with pentoxifylline, alone and in combination with nitric oxide synthase or cyclooxygenase inhibition, on (A) sciatic composite endoneurial blood flow, (B) composite vascular conductance (VC) and (C) the proportion of endoneurial hydrogen clearance carried by the nutritive flow component. Group details are given in the legend to Figure 2. Data are mean ± SEM.

to several unrelated treatments, including aldose reductase inhibitors, a vasodilator and an antioxidant, has been previously observed in diabetic rats (Cameron *et al.*, 1994b, 1989, 1994c; Nagamatsu *et al.*, 1995). Pentoxifylline can act as a phosphodiesterase inhibitor, and a recent study showed that 4 weeks of treatment with a high dose of the type III (cAMP) phophodiesterase inhibitor, cilostazol, prevented reductions in motor NCV by ~30% for tibial and ~60% for caudal nerve in diabetic rats (Kihara *et al.*, 1995). That degree of protection is in reasonable agreement with the magnitude of correction of proximal sciatic nerve motor NCV seen with pentoxifylline treatment in this study.

Sciatic endoneurial nutritive blood flow was increased by pentoxifylline, roughly in proportion to the effect on motor conduction. This provides further support for a vascular etiology of nerve dysfunction in diabetes. High-dose cilostazol treatment approximately halved the nutritive endoneurial blood flow deficit in diabetic rats (Kihara *et al.*, 1995). While pentoxifylline, like cilostazol, has phosphodiesterase inhibitor properties, it also has several other actions that may be relevant for diabetic neuropathy. Thus, pentoxifylline reduces cytokine formation by phagocytes and blood vessels, particularly that of tumor necrosis factor (Bernard *et al.*, 1995; Mandell, 1995). The latter can evoke several deleterious vascular changes in diabetes relevant to vasa nervorum dysfunction. Tumor necrosis factor can activate protein kinase C (Deisher *et al.*, 1993) and stimulate nuclear factor $\kappa$B in endothelial cells (Tozawa *et al.*, 1995). This transcription factor causes several effects including elevated endothelin-1 synthesis, decreased NO production, and altered expression of adhesion molecules and increased cell-endothelium interaction (Collins, 1993; Rubanyi and Polokoff, 1994; Limb *et al.*, 1996; Bierhaus *et al.*, 1997). Circulating levels of tumor necrosis factor are elevated in diabetic rats, and this may be prevented by treatment with *N*-acetyl-L-cysteine, which also restores endothelial function, endoneurial blood flow and NCV (Archibald *et al.*, 1996; Love *et al.*, 1996a; Sagara *et al.*, 1996).

Pentoxifylline has also been identified as a scavenger of hydroxyl (but not superoxide)

radicals *in vitro* (Freitas and Filipe, 1995) over a concentration range relevant to the dose used in this *in vivo* study. The production of reactive oxygen species is increased by diabetes and endogenous antioxidant defense mechanisms in nerve are compromised (Cameron *et al.*, 1999; Nagamatsu *et al.*, 1995; Nickander *et al.*, 1994). Hydroxyl radicals are short-lived but highly reactive and can be formed by transition metal catalyzed processes such as the Fenton reaction, which are relevant to diabetes (Cameron and Cotter, 1995a). Another source of hydroxyl radicals is the breakdown of peroxynitrite (Beckman *et al.*, 1990), formed from the reaction between NO and superoxide. This process probably contributes to endothelial dysfunction (Pieper *et al.*, 1993) and neurovascular deficits (Kihara and Low, 1995; Maxfield *et al.*, 1997) in experimental diabetes. While there have not been any reports on the effects of specific hydroxyl radical scavengers on nerve function, transition metal chelators are effective in restoring nerve blood flow, NCV and regenerative capacity in experimental models of diabetic neuropathy (Cameron and Cotter, 1995a; Love *et al.*, 1996b).

The platelet anti-aggregatory effect of pentoxifylline is likely to depend on inhibition of platelet phosphodiesterase, allowing elevated cAMP levels to inhibit the formation of thromboxane $A_2$ (Ward and Clissold, 1987). However, this requires relatively high doses that may not have been attained in this *in vivo* experiment. In addition, use of specific thromboxane $A_2$ receptor/thromboxane synthase antagonist treatment only has a modest effect ($\sim$16% correction) on motor NCV in diabetic rats (Dines *et al.*, 1996). Although the rheological/anti-platelet actions of pentoxifylline may be contributory; alone they are probably not sufficient to account for the effects on neurovascular function in diabetic rats. The improvements in NCV and blood flow with pentoxifylline were attenuated by co-treatment with the cyclooxygenase inhibitor, flurbiprofen, but not by NO synthase inhibition. This suggests that the major action of pentoxifylline in these experiments depended upon cyclooxygenase products rather than NO. A vasa nervorum prostacyclin deficit contributes to reduced nerve blood flow in diabetic rats (Ward *et al.*, 1989). This appears to be caused primarily by a deficit in substrate availability, since it is corrected by treatment with $\omega$-6 essential fatty acids such as $\gamma$-linolenic or arachidonic acid (Cameron and Cotter, 1999). Prostacyclin promotes vascular smooth muscle relaxation *via* a cAMP-dependent mechanism. It is plausible, therefore, that a major effect of pentoxifylline is to reduce cAMP breakdown *via* a phosphodiesterase inhibitor action, thus potentiating the vasorelaxant effects of endogenous prostacyclin. This would at least partially compensate for reduced vasa nervorum prostacyclin synthesis, and is in accord with the finding that prostacyclin analogs correct NCV and blood flow deficits in diabetic rats (Cotter *et al.*, 1993; Hotta *et al.*, 1996).

The resistance of pentoxifylline treatment to NO synthase inhibition sets it apart from several other drugs that improve endoneurial perfusion and NCV in diabetic rats. These include aldose reductase inhibitors (Cameron *et al.*, 1996; Stevens *et al.*, 1994), aminoguanidine (Cameron and Cotter, 1996b), antioxidants (Cameron and Cotter, 1995b) and protein kinase C inhibitors (Cameron *et al.*, 1999). These drugs all attenuate the development of defective NO-mediated endothelium-dependent vasorelaxation in diabetes (Cameron and Cotter, 1994a; Archibald *et al.*, 1996). However, the effects of aldose reductase inhibitors are also opposed by flurbiprofen, perhaps because they moderately improve endothelial production of prostacyclin and other prostanoids and act on the NO system (Law and King, 1990; Wakasugi *et al.*, 1991).

Pentoxifylline treatment caused a change in the pattern of endoneurial blood flow in diabetic rats. While there was an overall increase in flow, including the nutritive capillary component, non-nutritive flow was emphasized. This suggests that there was a relative increase in arteriovenous shunting and is compatible with the notion that pentoxifylline potentiates the vascular actions of prostanoids. Evening primrose oil treatment, that supplies a high dose of $\gamma$-linolenic acid promoting prostanoid synthesis, increases nerve blood flow and has a particularly marked action on the non-nutritive component (Cameron and Cotter, 1994b). Conversely, flurbiprofen treatment of diabetic rats had the opposite effect, reducing non-nutritive more than the nutritive flow (Cameron *et al.*, 1996). On the other hand, aldose reductase inhibitors favor nutritive perfusion (Cameron *et al.*, 1994b), although they can increase prostanoid production. It is possible that the different drug effects on endoneurial blood flow depends on how they alter the balance between prostanoid and NO actions on vasa nervorum.

In conclusion, pentoxifylline partially corrected the endoneurial blood flow deficit, and improved NCV in diabetic rats. These neurovascular effects probably depend in large part on a potentiation of prostanoid-mediated vasodilator and rheological actions. Pentoxifylline is used in the treatment of peripheral vascular disease (Ward and Clissold, 1987), and it is possible that it may also prove useful in the treatment of diabetic neuropathy.

### Acknowledgments

This research was supported in part by a grant from the British Diabetic Association.

### References

Adams, J. G. Jr., Dhar, A., Shukla, S. D. and Silver, D. (1995) Effect of pentoxifylline on tissue injury and platelet-activating factor production during ischemia-reperfusion injury. *J. Vasc. Surg.*, **21**, 742–748.

Ambrus, J. L., Stadler, S. and Kulaylat, M. (1995) Hemorrheologic effects of metabolites of pentoxifylline (Trental). *J. Med.*, **26**, 65–75.

Archibald, V., Cotter, M. A., Keegan, A. and Cameron, N. E. (1996) Contraction and relaxation of aortas from diabetic rats; effects of chronic anti-oxidant and aminoguanidine treatments. *Naunyn-Schmiedebergs Arch. Pharmacol.*, **353**, 584–591.

Beckman, J. S., Beckman, T. W., Chen, J., Marshall, P. A. and Freeman, B. A. (1990) Apparent hydroxyl radical production by peroxynitrite: implications for endothelial injury from nitric oxide and superoxide. *Proc. Natl. Acad. Sci. USA*, **87**, 1620–1624.

Bernard, C., Barnier, P., Merval, R., Esposito, B. and Tedgui, A. (1995) Pentoxifylline selectivity inhibits tumor necrosis factor synthesis in the arterial wall. *J. Cardiovasc. Pharmacol.*, **25**(suppl. 2), S30–S33.

Bierhaus, A., Chevion, S., Chevion, M., Hofmann, M., Quehenberger, P., Illmer, T., Luther, Y., Berentshtein, E., Tritschler, H., Müller, M., Wahl, P., Ziegler, R. and Nawroth, P. P. (1997) Advanced glycation end product-induced activation of NF-$\kappa$B is suppressed by $\alpha$-lipoic acid in cultured endothelial cells. *Diabetes*, **46**, 1481–1490.

Cameron, N. E. and Cotter, M. A. (1994a) The relationship of vascular changes to metabolic factors in diabetes mellitus and their role in the development of peripheral nerve complications. *Diabetes Metab. Rev.*, **10**, 189–224.

Cameron, N. E. and Cotter, M. A. (1994b) Effects of evening primrose oil treatment on sciatic nerve blood flow and endoneurial oxygen tension in streptozotocin-diabetic rats. *Acta Diabetologica*, **31**, 220–225.

Cameron, N. E. and Cotter, M. A. (1995a) Neurovascular deficits in diabetic rats: potential contribution of autoxidation and free radicals examined using transition metal chelating agents. *J. Clin. Invest.*, **96**, 1159–1163.

Cameron, N. E. and Cotter, M. A. (1995b) Reversal of peripheral nerve conduction and perfusion deficits by the free radical scavenger, BM15.0639, in diabetic rats. *Naunyn-Schmiedeberg's Arch. Pharmacol.*, **352**, 685–690.

Cameron, N. E. and Cotter, M. A. (1996a) Effects of a nonpeptide endothelin-1 $ET_A$ antagonist on neurovascular function in diabetic rats: Interaction with the renin-angiotensin system. *J. Pharmacol. Exp. Ther.*, **278**, 1262–1268.

Cameron, N. E. and Cotter, M. A. (1996b) Rapid reversal by aminoguanidine of the neurovascular effects of diabetes in rats: modulation by nitric oxide synthase inhibition. *Metabolism*, **45**, 1147–1152.

Cameron, N. E. and Cotter, M. A. (1999) The role of linolenic acid in diabetic polyneuropathy. In: Dyck, P. J. and Thomas, P. K. (Eds.) *Diabetic Neuropathy*, 2nd edn., (Philadelphia: Saunders), pp. 359–367.

Cameron, N. E., Cotter, M. A. and Robertson, S. (1989) The effect of aldose reductase inhibition on the pattern of nerve conduction deficits in diabetic rats. *Q. J. Exp. Physiol.*, **74**, 917–926.

Cameron, N. E., Cotter, M. A. and Low, P. A. (1991) Nerve blood flow in early experimental diabetes in rats: relation to conduction deficits. *Am. J. Physiol.*, **261**, E1–E8.

Cameron, N. E., Cotter, M. A., Dines, K. C. and Maxfield, E. K. (1993) Pharmacological manipulation of vascular endothelium in non-diabetic and streptozotocin-diabetic rats: effects on nerve conduction, hypoxic resistance and endoneurial capillarization. *Diabetologia*, **36**, 516–522.

Cameron, N. E., Cotter, M. A., Archibald, V., Dines, K. C. and Maxfield, E. K. (1994a) Anti-oxidant and pro-oxidant effects on nerve conduction velocity, endoneurial blood flow and oxygen tension in non-diabetic and streptozotocin-diabetic rats. *Diabetologia*, **37**, 449–459.

Cameron, N. E., Cotter, M. A., Dines, K. C., Maxfield, E. K., Carey, F. and Mirrlees, D. (1994b) Aldose reductase inhibition, nerve perfusion, oxygenation and function in streptozotocin-diabetic rats: dose-response considerations and independence from a myoinositol mechanism. *Diabetologia*, **37**, 651–663.

Cameron, N. E., Dines, K. C. and Cotter, M. A. (1994c) The potential contribution of endothelin-1 to neurovascular abnormalities in streptozotocin-diabetic rats. *Diabetologia*, **37**, 1209–1215.

Cameron, N. E., Cotter, M. A. and Hohman, T. C. (1996) Interactions between essential fatty acid, prostanoid, polyol pathway and nitric oxide mechanisms in the neurovascular deficit of diabetic rats. *Diabetologia*, **39**, 172–182.

Cameron, N. E., Cotter, M. A., Jack, A. M., Basso, M. D. and Hohman, T. C. (1999) Protein kinase C effects on nerve function, perfusion, $Na^+$, $K^+$-ATPase activity and glutathione content in diabetic rats. *Diabetologia*, **42**, 1120–1130.

Ceriello, A. (1993) Coagulation activation in diabetes mellitus: the role of hyperglycaemia and therapeutic prospects. *Diabetologia*, **36**, 1119–1125.

Collins, T. (1993) Endothelial nuclear factor-$\kappa$B and the initiation of the atherosclerotic lesion. *Lab. Invest.*, **68**, 499–508.

Cotter, M. A., Flint, H. and Cameron, N. E. (1997) Cyclooxygenase-dependent effect of pentoxifylline on neurovascular function in diabetic rats. *Diabetologia*, **40** (suppl. 1), A549.

Cotter, M. A., Dines, K. C. and Cameron, N. E. (1993) Prevention and reversal of motor and sensory peripheralnerve conduction abnormalities in streptoztocin-diabetic rats by the prostacyclin analogue iloprost. *Naunyn-Schmiedeberg's Arch. Pharmacol.*, **347**, 534–540.

Day, T. J., Lagerlund, T. D. and Low, P. A. (1989) Analysis of $H_2$ clearance curves used to measure blood flow in rat sciatic nerve. *J. Physiol.*, **414**, 35–54.

Deisher, T. A., Sato, T. T., Pohlman, T. H. and Harlan, J. M. (1993) A protein kinase C agonist, selective for the beta I isozyme, induces E-selectin and VCAM-1 expression on HUVEC but does not translocate PKC. *Biochem. Biophys. Res. Commun.*, **193**, 1283–1290.

Dines, K. C., Cotter, M. A. and Cameron, N. E. (1996) Effectiveness of natural oils as sources of $\gamma$-linolenic acid to correct peripheral nerve conduction velocity abnormalities in diabetic rats: modulation by thromboxane $A_2$ inhibition. *Prostaglandins Leukot. Essent. Fatty Acids*, **55**, 159–165.

Freitas, J. P. and Filipe, P. M. (1995) Pentoxifylline. A hydroxyl radical scavenger. *Biol. Trace Element Res.*, **47**, 307–311.

Hansen, P. R. (1994) *In vitro* studies on responses to pentoxifylline and aminophylline of rat mesenteric resistance vessels. *Eur. J. Pharmacol.*, **261**, 105–110.

Hotta, N., Koh, N., Sakakibara, F., Nakamura, J., Kakuta, H., Fukasawa, H. and Sakamoto, N. (1996) Effects of beraprost sodium and insulin on the electroretinogram, nerve conduction and nerve blood flow in rats with streptozotocin-induced diabetes. *Diabetes*, **45**, 361–366.

Kaputlu, I. and Sadan, G. (1994) Pentoxifylline-induced vasodilatation is not endothelium-dependent in rabbit aorta. *J. Basic Clin. Physiol. Pharmacol.*, **5**, 295–304.

Kihara, M. and Low, P. A. (1995) Impaired vasoreactivity to nitric oxide in experimental diabetic neuropathy. *Exp. Neurol.*, **132**, 180–185.

Kihara, M., Schmelzer, J. D. and Low, P. A. (1995) Effect of cilostazol on experimental diabetic neuropathy in the rat. *Diabetologia*, **38**, 914–918.

Law, S. C. and King, R. G. (1990) Effects of ponalrestat on depressor responses to arachidonic acid in streptozotocin-diabetic rats. *Gen. Pharmacol.*, **21**, 135–139.

Limb, G. A., Chignell, A. H., Green, W., LeRoy, F. and Dumonde, D. C. (1996) Distribution of TNF alpha and its reactive vascular adhesion molecules in fibrovascular membranes of proliferative diabetic retinopathy. *Br. J. Ophthalmol.*, **80**, 168–173.

Love, A., Cameron, N. E. and Cotter, M. A. (1996a) Effects of the sulphydryl donor, *N*-acetyl-L-cysteine on nerve conduction, perfusion, maturation and regeneration following damage in streptozotocin-diabetic rats. *Eur. J. Clin. Invest.*, **26**, 698–706.

Love, A., Cotter, M. A. and Cameron, N. E. (1996b) Nerve function and regeneration in diabetic and galactosaemic rats: antioxidant and metal chelator effects. *Eur. J. Pharmacol.*, **314**, 33–39.

Mandell, G. L. (1995) Cytokines, phagocytes, and pentoxifylline. *J. Cardiovasc. Pharmacol.*, **25**(suppl. 2), S20–S22.

Maxfield, E. K., Love, A., Cotter, M. A. and Cameron, N. E. (1995) Nerve function and regeneration in diabetic rats: effects of ZD-7155, an $AT_1$ receptor antagonist. *Am. J. Physiol.*, **269**, E530–E537.

Maxfield, E. K., Cameron, N. E. and Cotter, M. A. (1997) Effect of diabetes on reactivity of sciatic vasa nervorum in rats. *J. Diabet. Complications*, **11**, 47–55.

Nagamatsu, M., Nickander, K. K., Schmelzer, J. D., Raya, A., Wittrock, A., Tritschler, H. and Low, P. A. (1995) Lipoic acid improves nerve blood flow, reduces oxidative stress and improves distal nerve conduction in experimental diabetic neuropathy. *Diabetes Care*, **18**, 1160–1167.

Nickander, K. K., Schmelzer, J. D., Rohwer, D. A. and Low, P. A. (1994) Effects of $\alpha$-tocopherol deficiency on indices of oxidative stress in normal and diabetic peripheral nerve. *J. Neurol. Sci.*, **126**, 6–14.

Pieper, G. M., Langenstroer, P. and Gross, G. J. (1993) Hydroxyl radicals mediate injury to endothelium-dependent relaxation in diabetic rat. *Molec. Cell Biochem.*, **122**, 139–145.

Rubanyi, G. M. and Polokoff, M. A. (1994) Endothelins: molecular biology, biochemistry, pharmacology, physiology and pathophysiology. *Pharmacol. Rev.*, **46**, 325–415.

Sagara, M., Satoh, J., Wada, R., Yagihashi, S., Takahashi, K., Fukuzawa, M., Muto, G., Muto, Y. and Toyota, T. (1996) Inhibition of development of peripheral neuropathy in streptozotocin-induced diabetic rats with N-acetylcysteine. *Diabetologia*, **39**, 263–269.

Sima, A. A. F. and Sugimoto, K. (1999) Experimental diabetic neuropathy: an update. *Diabetologia*, **42**, 773–788.

Simpson, L. O. (1988) Altered blood rheology in the pathogenesis of diabetic and other neuropathies. *Muscle Nerve*, **11**, 725–744.

Stevens, M. J., Dananberg, J., Feldman, E. L., Lattimer, S. A., Kamijo, M., Thomas, T. P., Shindo, H., Sima, A. A. F. and Greene, D. A. (1994) The linked roles of nitric oxide, aldose reductase and ($Na^+$, $K^+$)-ATPase in the slowing of nerve conduction in the streptozotocin diabetic rat. *J. Clin. Invest.*, **94**, 853–859.

Tesfaye, S., Malik, R. and Ward, J. D. (1994) Vascular factors in diabetic neuropathy. *Diabetologia*, **37**, 847–854.

Tozawa, K., Sakurada, S., Kohri, K. and Okamoto, T. (1995) Effects of anti-nuclear factor kappa B reagents in blocking adhesion of human cancer cells to vascular endothelial cells. *Cancer Res.*, **55**, 4162–4167.

Tuck, R. R., Schmelzer, J. D. and Low, P. A. (1984) Endoneurial blood flow and oxygen tension in the sciatic nerves of rats with experimental diabetic neuropathy. *Brain*, **107**, 935–950.

Wakasugi, M., Noguchi, T., Inoue, M., Tawata, M., Shindo, H. and Onaya, T. (1991) Effects of aldose reductase inhibitors on prostacyclin ($PGI_2$) synthesis by aortic rings from rats with streptozotocin-induced diabetes. *Prostaglandins Leukot. Essent. Fatty Acids*, **44**, 233–236.

Ward, A. and Clissold, S. P. (1987) Pentoxifylline. A review of its pharmacodynamic and pharmacokinetic properties and its therapeutic efficacy. *Drugs*, **34**, 50–97.

Ward, K. K., Low, P. A., Schmelzer, J. D. and Zochodne, D. W. (1989) Prostacyclin and noradrenaline in peripheral nerve of chronic experimental diabetes in rats. *Brain*, **112**, 197–208.

